# Cannabidiol in autism: Clinical pharmacology priorities for trial design

**DOI:** 10.1002/bcp.70400

**Published:** 2025-11-29

**Authors:** Gislei Frota Aragão, Carla Barbosa Brandão, José Evaldo Leandro Júnior

**Affiliations:** ^1^ Medicine Course, Health Sciences Center State University of Ceará Fortaleza Brazil; ^2^ Faculty of Medicine, Department of Physiology and Pharmacology, Drug Research and Development Center Federal University of Ceará Fortaleza Brazil

Recent randomized controlled trials of purified cannabidiol (CBD) for severe behavioural symptoms in autistic boys mark a significant advance in cannabinoid research for autism spectrum disorder (ASD).^1^ While these studies employ rigorous methodologies, including pharmacokinetic monitoring and double‐blind designs, their null findings prompt a re‐evaluation of key clinical pharmacology principles to optimize future trial designs. Three critical factors, outcome measure selection, dose–response relationships and treatment duration, warrant particular attention, alongside considerations of pharmacokinetic interactions and placebo‐response mitigation.

Outcome selection is the first high‐leverage decision. Diagnostic instruments such as the Autism Diagnostic Observation Schedule, Second Edition (ADOS‐2) provide calibrated severity metrics valuable for case definition and phenotyping, yet they were not built to capture short‐term treatment response. Their longitudinal properties in naturalistic cohorts show relative stability and only modest associations with functional change, which limits responsiveness to pharmacological effects over weeks rather than years.^2^ Trials should therefore anchor primary endpoints in validated, treatment‐responsive instruments such as the Aberrant Behavior Checklist‐Irritability (ABC‐I) or Clinical Global Impression‐Improvement (CGI‐I) and use ADOS‐2, when needed, as a descriptive or exploratory measure rather than as a primary outcome.

Dose is the second determinant. Human data indicate that CBD can follow an inverted U‐shaped dose–response for certain neurobehavioural outcomes, with intermediate doses producing stronger effects than very low or very high doses.^3^ In epilepsy, fixed high doses around 20 mg/kg/day are supported by safety and efficacy data for seizure reduction and have become a default ceiling in other indications.^4^ However, translating that ceiling wholesale to ASD behavioural endpoints risks dosing above the optimal window for anxiolytic or modulatory effects, where sedation, interaction liabilities or tachyphylaxis may reduce the measurable benefit. Although observational designs cannot establish causality, they strengthen biological plausibility for dose finding in randomized settings. Consequently, ASD trials should incorporate explicit dose ranging, preferably adaptive designs that test at least three dose levels spanning the hypothesised optimum, instead of single, fixed high‐dose regimens.

Exposure time is the third lever. Several CBD mechanisms relevant to behaviour, including 5‐HT1A modulation, GABAergic effects, immune signalling and synaptic plasticity, are unlikely to plateau within a few weeks. In a randomized, placebo‐controlled trial for anxiety using a nanodispersible oral CBD solution, clinical benefit emerged progressively from Week 2 and stabilized only around Weeks 9 to 12.^5^ While the indication differs, the temporal pattern supports a conservative stance: 4 weeks of active treatment may be underpowered to detect the full neuroadaptive effect profile on ASD‐related behaviours. For ASD trials, a minimum of 10–12 weeks of stable dosing is a defensible starting point for primary endpoint assessment, with earlier checkpoints reserved for safety and trajectory exploration.^5^

Pharmacokinetics and drug–drug interactions are the fourth constraint. CBD is an inhibitor of CYP3A4 and CYP2C19 and interacts with additional transporters and enzymes.^6^ Concomitant medications commonly used in ASD (such as antipsychotics, alpha‐2 agonists, SSRIs, anticonvulsants and stimulants) can alter CBD exposure, and conversely, CBD can inhibit their metabolism, increasing plasma concentrations and the likelihood of adverse effects that may be misinterpreted as efficacy or tolerability signals.^7^ Failure to manage this bidirectional complexity prospectively can both attenuate signal and inflate variability. Trials should institute standardized rules for comedications: either exclude specific classes with high interaction potential or stratify randomisation and analyse subgroups by comedication class. Sparse PK sampling with population modelling can quantify exposure variability, and integrating exposure–response analyses improves the credibility of negative findings by showing whether nonresponse co‐occurred with subtherapeutic concentrations. Furthermore, integrating pharmacokinetic–pharmacodynamic (PK‐PD) work strengthens interpretability: sparse PK at Weeks 2, 6 and 12 with population modelling to estimate AUC and trough concentration (*C*_min_); covariate handling of comedications; exposure–response analyses such as *E*_max_ on ABC‐I or CGI‐I; definition of a target exposure range consistent with an intermediate efficacy window; and avoidance of rapid escalation to 20 mg/kg/day without dose finding.^3^, ^4^, ^5^, ^6^ Safety monitoring should include periodic hepatic panels (ALT/AST) and targeted sampling for interaction signals when comedications change, consistent with established safety experience and metabolic liabilities.^4^, ^6^ Null results with subtherapeutic levels suggest an exposure problem, and adequate levels without response support a lack of effect on the chosen endpoint.^3^, ^4^, ^5^, ^6^

Expectancy effects are the fifth challenge. Paediatric neurodevelopmental trials are susceptible to placebo responses that reflect caregiver expectations, contact intensity with research staff and imperfect blinding. Simple, participant‐focused psychoeducation about placebo probability and trial uncertainty can reduce expectancy‐driven responses, as shown in psychiatric settings.^8^ Measuring expectancy and perceived treatment assignment at baseline and serially allows statistical adjustment and provides an audit trail for blinding integrity. Equally important is protocol discipline that limits non‐essential supportive interactions that can amplify expectancy.^8^ A brief single‐blind placebo run‐in may be considered to identify high placebo responders, though it can introduce selection bias and requires careful justification.

Observational evidence deserves cautious, targeted use. A large real‐world cohort with CBD‐rich or full‐spectrum preparations reported improvements in irritability, sleep, anxiety and social engagement with acceptable tolerability, and an open‐label study found similar patterns with CBD‐rich cannabis.^9^, ^10^ These data are not substitutes for randomized evidence, but they can inform three practical choices: which domains matter most to families and should be prioritized as outcomes, which dose ranges warrant prospective testing and which adverse effects and interactions deserve proactive monitoring. Incorporating these lessons into randomized designs narrows the hypothesis space and increases the probability that a true effect, if present, will be captured.^9^, ^10^

Putting these threads together yields a clinical pharmacology template for ASD‐CBD trials. Begin with a dose‐finding stage that brackets the suspected optimum derived from human dose–response and real‐world titration patterns.^3^, ^9^ Use an adaptive design to allocate more participants to promising doses as the trial progresses while protecting against type I error. Such designs and sparse PK are more feasible in multicentre consortia with preplanned logistics and central laboratories. Ensure exposure sufficiency with at least 10–12 weeks of stable dosing before the primary endpoint, and embed sparse PK sampling to model exposure variability across comedication strata.^5^, ^6^ Longer exposure increases attrition risk; use digital reminders and flexible visit windows to support adherence. Select a single, treatment‐responsive primary outcome aligned with the symptom domain most likely to change over that interval, often irritability or sleep and relegate broad diagnostic metrics to secondary, exploratory status.^2^ Preregister a limited set of secondary outcomes with clear directionality and clinical meaning to avoid multiplicity and selective emphasis. Implement expectancy‐mitigation procedures, measure perceived assignment and prespecify sensitivity analyses that adjust for expectancy.^8^ Finally, report negative trials with supporting pharmacokinetic and exposure–response data, to help the field distinguish between true lack of pharmacological effect and insensitivity due to subtherapeutic exposure or design limitations.^5^, ^6^, ^8^ Include girls and prespecify sex‐stratified analyses to address phenotype differences and potential PK‐PD variation by sex.

Such a template also accommodates formulation questions that matter in practice. Purified CBD removes confounders but may not replicate outcomes attributed to multicomponent extracts where terpenes and minor cannabinoids could modulate effect size or tolerability. Rather than pitting purified against full‐spectrum abstractly, an efficient path is staged: Establish whether purified CBD shows a clinically relevant effect under optimized conditions; if yes, test whether adding defined minor components produces additive or synergistic benefit on prespecified outcomes; if not, investigate whether alternative targets or formulations better match ASD pathophysiology. Either way, careful PK‐PD work, dose–response characterization and outcome sensitivity remain decisive.

The ethical and practical stakes justify this rigour. Families seek relief for disruptive irritability, insomnia and anxiety that impair daily function. Clinicians need estimates not only of whether CBD works, but for whom, at what dose, over what time and under which comedications. Regulators and payers require designs that separate expectancy‐driven improvement from pharmacological effect and that report harms transparently. The clinical pharmacology programme outlined here is realistic within standard trial budgets and timelines, and it answers the questions each stakeholder asks.

Feasibility and cost. Pragmatic strategies can reduce type II error and clarify exposure–response relationships without disproportionate budget inflation. Sparse pharmacokinetic sampling combined with exposure–response modelling, adaptive designs and testing only a few doses within the optimal range can provide robust information at relatively low incremental cost. Coupled with placebo mitigation approaches (short run‐in, expectancy assessment and statistical adjustment) and decentralized trial elements (electronic patient‐reported outcomes, remote visits), these measures preserve internal validity, reduce operational burden and maintain feasibility for sponsors, bridging methodological rigour with practical trial realities.

In summary, the probability of detecting a true CBD effect on ASD‐related behaviours depends more on a handful of design decisions than on any single molecule property. Trials that choose responsive outcomes, map dose–response, allow sufficient exposure time, manage pharmacokinetics and comedications and measure expectancy are more likely to yield results that are both scientifically credible and clinically useful.

## CONFLICT OF INTEREST STATEMENT

The authors declare no conflict of interest.
